# Utilization of Smartphone Applications by Anesthesia Providers

**DOI:** 10.1155/2018/8694357

**Published:** 2018-02-08

**Authors:** Michael S. Green, Johann J. Mathew, Archana Gundigi Venkatesh, Parmis Green, Rayhan Tariq

**Affiliations:** Department of Anesthesiology and Perioperative Medicine, Drexel University College of Medicine, 245 N. 15th Street, Suite 7502, MS 310, Philadelphia, PA 19102, USA

## Abstract

Health care-related apps provide valuable facts and have added a new dimension to knowledge sharing. The purpose of this study is to understand the pattern of utilization of mobile apps specifically created for anesthesia providers. Smartphone app stores were searched, and a survey was sent to 416 anesthesia providers at 136 anesthesiology residency programs querying specific facets of application use. Among respondents, 11.4% never used, 12.4% used less than once per month, 6.0% used once per month, 12.1% used 2-3 times per month, 13.6% used once per week, 21% used 2-3 times per week, and 23.5% used daily. Dosage/pharmaceutical apps were rated the highest as most useful. 24.6% of the participants would pay less than $2.00, 25.1% would pay $5.00, 30.3% would pay $5–$10.00, 9.6% would pay $10–$25.00, 5.1% would pay $25–$50.00, and 5.1% would pay more than $50.00 if an app saves 5–10 minutes per day or 30 minutes/week. The use of mobile phone apps is not limited to reiterating information from textbooks but provides opportunities to further the ever-changing field of anesthesiology. Our survey illustrates the convenience of apps for health care professionals. Providers must exercise caution when selecting apps to ensure best evidence-based medicine.

## 1. Introduction

Internet connectivity on the go has become a necessity for the millennial generation, and the use of the smartphone technology has helped keep people connected at all times. The concept of smartphones was introduced in the 1970s, but it took another 20 years prior to becoming available in the mainstream. From 2004 to 2007, there was a dramatic rise in smartphone usage. In 2007, Apple introduced the iPhone and Google the Android phone in 2008 [1], and with that ushered the meteoric rise of smartphones. Although, landline phones have been in use for decades, there has been an increasing trend towards “only wireless” phones in almost half of the American homes [2].

Smartphones can perform many of the common functions of a computer with the added advantage of “always being available” and maximum versatility, much like a Swiss Army knife. With a large screen and high-quality resolution, expandable memory, and a powerful processor, they are much like a handheld computer [3] and are touted to be the PCs of the future. This becomes critical for health care professionals who spend little time sitting in front of a computer terminal [4].

Health care-related apps provide valuable facts in different hospital settings, including the perioperative and intensive care unit (ICU) environment. Information regarding medications, symptoms of diseases, diagnosis, and dosage calculators is readily available at the physician's fingertips. In addition, these apps help health care professionals with tasks such as time management, health record maintenance and access, communications, consulting, monitoring, medical education, and training [1, 5–9]. The use of smartphones and apps has added a new dimension to how medical knowledge is shared and has altered the way medicine is practiced [10].

A variety of apps are available in the market: some are free and others paid, and it is believed that there are more than 100,000 health care-related medical apps [11]. The use of smartphone apps has been described in various specialties including orthopedics [12], dermatology [13], anesthesiology [14], neurosurgery [15], plastic surgery [16], urology [17], and infectious diseases [18]. However, no study to date has reviewed the use of anesthesia apps and how they can help integrate technology into clinical practice.

While it is easy to see why medical apps are popular among health care professionals (HCPs), the issue of reliability and quality still remains. In many of the health care-related applications, there was no information received from HCPs during the development leaving the expert medical input lacking in many cases.

The purpose of this study is to understand the pattern of utilization of mobile apps specifically created for anesthesia providers and trainees. The goal was to help in understanding, as well as guiding the future development of the mobile technology in anesthesiology.

## 2. Background and Current State of Anesthesiology Mobile Applications

The boosting prevalence of the health care-related smartphone technology generates a constantly increasing worldwide interest. Anesthesiology, as a profession, has been one of the first departments to adopt new technologies. In addition to the general medicine application, there are applications related to anesthesia in particular. There are numerous apps to choose from, each contributing to the various responsibilities of an anesthesiologist. The various apps in anesthesiology can be categorized as follows.

### 2.1. Medical Calculator Apps

These are one of the most widely used categories of apps. These apps, by using conventional formulas and equations, assist in calculating clinical scores and indices.

Remembering different formulas can be cumbersome, time-consuming, and increases the chances of error when working in a stressful environment. By entering the required parameters into the apps, the users can expect a quick and reliable result at the point of care [19]. These calculators have formulas for calculating various clinical scores and indices [10]; the input of patient's variables is the only requirement. Examples include calculation of body mass index (BMI) or determining the size of laryngeal mask airway for that patient with apps such as *Anesthesia Assistant* (abletFactory^©^).

### 2.2. Drug Reference Apps

There are several commercially available apps that comprise a continually updated drug database, providing reliable information on dosing, indications and contraindications, adverse reactions, interactions, and safety of commonly used medications. Also, their pill identifier feature enables one to find prescription drugs by imprint, shape, color, and scoring. These apps are very popular among physicians, with a reported usage of 90% [19]. Examples include *Medscape*, *Lexicomp*, *and Epocrates*.

*Epocrates* is one of the most commonly used drug reference apps that have been around since the Palm Pilot days [10, 20]. It has an extensive drug database, allowing users to search medications by generic or brand name. Its drug interaction feature enables users to list all the medicines the patient takes and then to look for any adverse interactions between them and thus contribute to patient safety [20].

### 2.3. Journal Apps

With most physicians spending a lot of time away from their desk, journal apps are a convenient way of staying updated with the latest scientific and medical research. Read by *QxMD*, *Docwise*, *Docphin*, and *BrowZine* are some of the commonly used journal apps. These apps function as a journal library, allowing the users to browse and read journals at one place. *Read* is linked to PubMed interface and reformatted to the mobile device, making for a convenient access to thousands of articles. *PubMed mobile* is a mobile-friendly interface with the same functions as the web page allowing for easy browsing. *Docphin* is yet another app that allows for easy access to journals. It requires the users to create an account initially. Institutional access permits access to PDFs. The app is linked to Dropbox, enabling files to be saved for later reading [21]. In addition, journals like *Anesthesia & Analgesia*, *Anesthesia*, and the *New England Journal of Medicine* have a mobile version which allows the reader to access their journals anytime.

### 2.4. Textbooks/References

These apps have the advantage of having volumes of textbooks converted to a mobile version, thus allowing for quick and immediate information. These apps can be updated regularly, thus allowing the subscriber to avoid buying newer editions. This also provides point of care access to reference textbooks, which would be otherwise impractical to carry in the operating room. A majority of textbooks these days come with an online and mobile app access to the contents of the books. For example, the Lippincott Williams & Wilkins publisher (*Wolters Kluwer^©^2017, Philadelphia, PA, USA*) provides a complementary mobile app access to majority of its new medical textbooks via the *Inkling* app (*2009–2016 Inkling Systems Inc.^©^*)

In context of anesthesiology, the mobile apps, in addition to providing useful readily available information, can also interact with the users. This interactive experience can be catered into simulation experience for the anesthesia providers. *iLarynx* is an interactive simulation that uses real-time technology to allow the clinician to practice fiberoptic laryngoscopy and endoscopy under a variety of conditions. Double lumen uses real bronchoscopic video images that the program retrieves as the user manipulates a virtual bronchoscope. The user chooses how he/she wants to move the scope, double-lumen tube, or blocker in an attempt to achieve the correct position. *iCPR* (D-Sign S.r.l.^©^) is another such app that uses the built-in accelerometer to detect chest compressions and provide feedback to the operator14. Similarly, *Airway EX* (2017 Level Ex Inc.^©^) allows anesthesia providers to practice their intubation skills using augmented reality.

Interestingly, anesthesia apps have found their way among the pediatric age group as well. In a study among the Korean children undergoing surgery, behavioral intervention program with a smartphone application was used as an alternative to premedication with a positive effect. This may allow the use of premedication at a low dose, if not getting rid of it all together in the future [22].

## 3. Methods

This study was aimed at surveying the pattern of anesthesiology apps used among anesthesia providers in the United States. Anesthesiology apps are currently available in the app store across different operating platforms. The Apple app store, Google play store, and Windows store were searched for “Anesthesia,” “Anesthesiology,” and “Airway.” The listed apps from each operating system were reviewed for last update, ratings from users, cost of the app, professional involvement of developers in the medical field, and the categories of different anesthesiology apps. Those with no reference to anesthesia or anesthesiology, non-English apps, and “lite-versions” of apps were excluded from the search. Also, we did not search specifically apps related to pain medicine.

In addition, an online survey was sent out to the Program Directors of 136 Accreditation Council for Graduate Medical Education- (ACGME-) accredited anesthesiology programs in USA using a hyperlink. It was requested that the survey be distributed to the anesthesiology attendings, residents, fellows, certified registered nurse anesthetists (CRNAs), and student RNAs (SRNAs). Institutional Review Board (IRB) approval was obtained for the study design, protocol, and survey questions. The survey consisted of nine questions (Table 1) and required an approximate duration of two minutes to complete. 416 complete responses were obtained, and the data were held anonymous. The data were analyzed utilizing SPSS version 12.

## 4. Results

The survey examined various mobile apps in the field of anesthesiology in the *Google play store*, *Apple store*, and *Windows store*. The apps were then divided into the following eight categories: journal apps, patient education, airway management simulators, dosage and pharmaceutical apps/medical calculators, professional society apps/conference adjuncts, textbooks/references, board review, and other educational apps (Table 2).

In the Apple play store, the search for anesthesiology and airway apps yielded 293 mobile applications, out of which 161 were found to be related to anesthesiology. The remaining apps were excluded. Out of 161 related apps, 5 were related to journals, 5 were airway management simulators, 30 includes both medical calculators and pharmacological dosage calculators, 11 apps were related to professional society apps/conference adjuncts, 69 were related to textbooks/references, 27 apps were related to board review, and 14 were related to other educational apps.

In the Google play store, a search for anesthesiology and airway yielded 250 mobile applications. 141 apps were related to anesthesiology, and the others were excluded. Out of 150, one was journal related, two were patient education, one was an airway management simulator, 20 were medical calculators and dosage/pharmaceutical apps, 10 were professional society apps/conference adjuncts, 57 were related to textbooks/references, 22 were related to board review, 9 were networking, scheduling, and patient safety apps combined, and 28 apps were related to other educational activities.

In the Windows store, a search for anesthesiology apps yielded 6 apps. Out of the 6, two were related to conference apps, one was related to board review, one was related to textbooks/reference, one was a medical calculator, and one was classified as other educational app. It is worth mentioning that Nokia and BlackBerry (latest devices like *BlackBerry Priv* and *BlackBerry DTEK50*) use Android operating system and hence run the same apps as the Google play store.

The second part of the survey focused on the utilization of anesthesiology apps among practitioners, including anesthesiology residents, anesthesiology fellows, certified registered nurse anesthetists (CRNA), SRNAs, and anesthesiology attending staff. A total of 416 people participated in the survey. Among the participants, the majority of them were attending anesthesiologists constituting 38.2% (*n* = 157). Others include anesthesiology residents which constitute 36.5% (*n* = 149), anesthesiology fellows constitute 2.0% (*n* = 8), CRNAs constitute 23.1% (*n* = 95), and SRNAs constitute 0.2% (*n* = 1).

**Figure 1:**
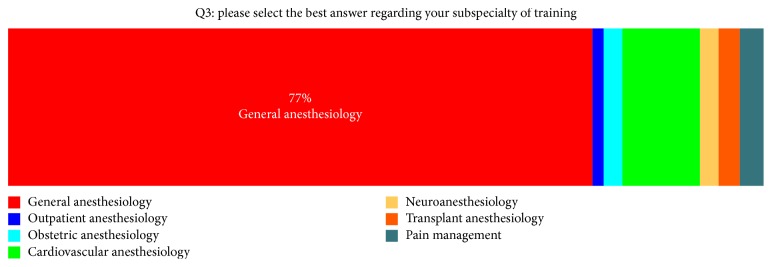
Specialty of the responding anesthesia providers.

Among the participants, 77.2% (*n* = 309) were practicing general anesthesiology, 10.3% (*n* = 41) were cardiovascular anesthesiologists, 1.5% (*n* = 6) were practicing outpatient anesthesiology, 2.5% (*n* = 10) were practicing obstetric anesthesiology, 2.5% (*n* = 10) were practicing neuroanesthesiology, 2.7% (*n* = 11) were practicing transplant anesthesiology, and 3.2% (*n* = 13) were practicing pain management (Figure 1). Among the survey participants, 99.3% (*n* = 410) were using smartphones and 0.7% (*n* = 03) of the participants were not using the smartphones. Among smartphone users, 18.1% (*n* = 74) were using Android, 81.7% (*n* = 334) were using iOS, 0.2% (*n* = 1) were using Windows mobile OS, and none of the participants were using the mobile phones utilizing BlackBerry OS, Symbian OS, or Palm OS.

Among the participants, 11.4% (*n* = 45) never used smartphone apps related to anesthesiology, 12.4% (*n* = 49) were using less than once per month, 6.1% (*n* = 24) were using once per month, 12.1% (*n* = 48) were using 2-3 times per month, 13.7% (*n* = 54) were using once per week, 20.9% (*n* = 83) were using 2-3 times per week, and 23.4% (*n* = 93) were using daily (Figure 1).

**Figure 3:**
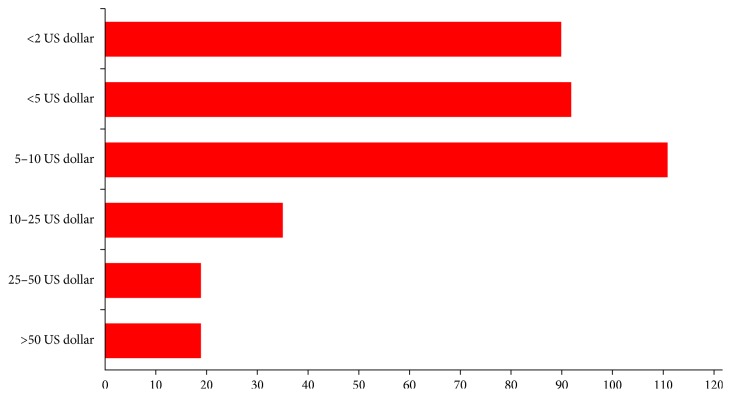
How much would you be willing to pay for an app that saves you 5–10 minutes/day or 30 minutes/week in your clinical practice?

In addition, the survey had questions related to how much participants were willing to pay for an app if it saves 5–10 minutes per day or up to 30 minutes/week (Figure 3). 24.6% (*n* = 90) of the participants were only willing to pay less than $2.00, 25.1% (*n* = 92) were willing to pay up to $5.00, 30.3% (*n* = 111) were willing to pay $5–$10.00, 9.6% (*n* = 35) were willing to pay $10–$25.00, 5.2% (*n* = 19) were willing to pay $25–$50.00, and 5.2% (*n* = 19) were willing to pay more than $50.00 (Figure 3). Finally, 84.1% (*n* = 308) were interested in newer apps in anesthesiology, and 15.9% (*n* = 58) were not interested in new anesthesia-related apps.

**Figure 4:**
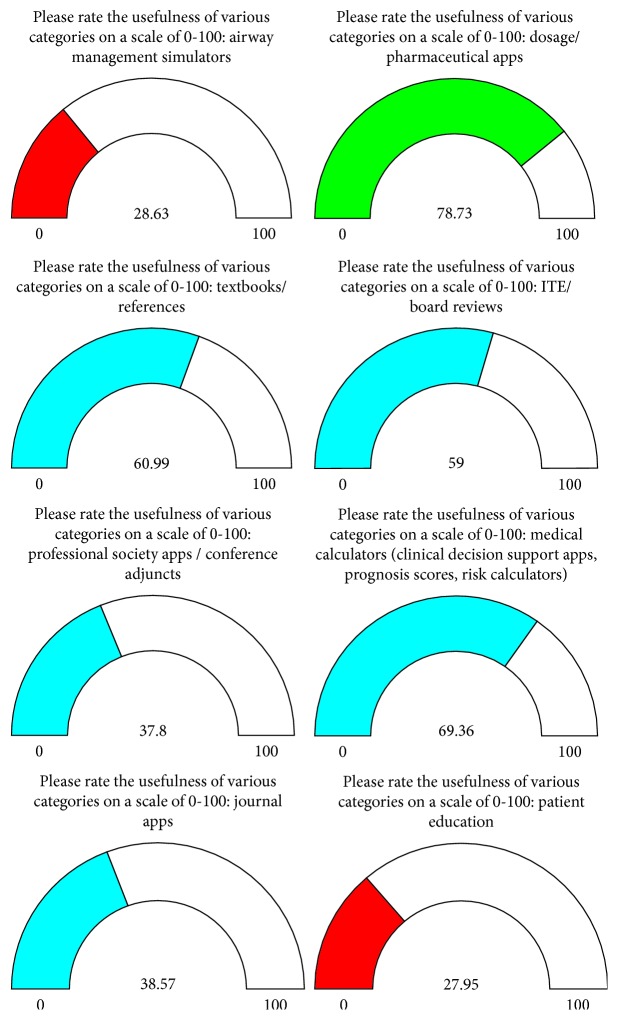
Usefulness of various anesthesiology applications as accessed by respondents.

**Figure 5:**
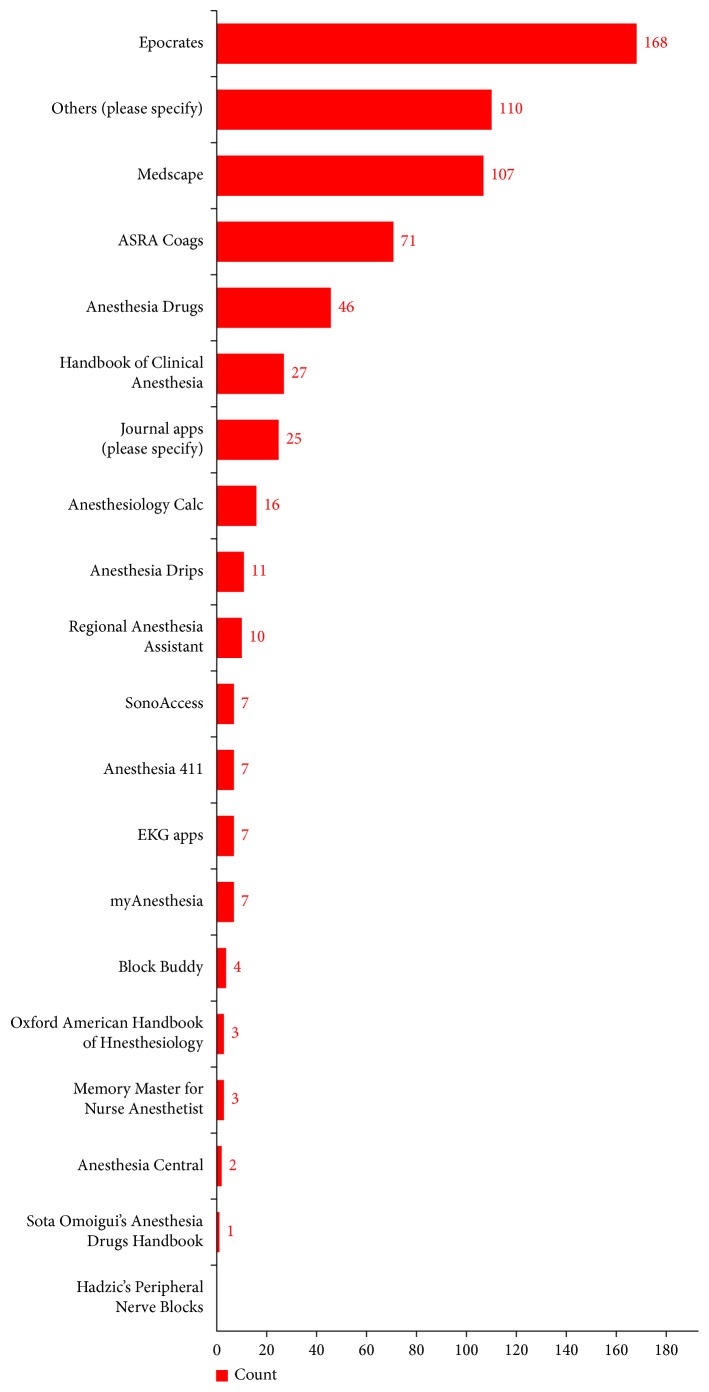
Most commonly used mobile applications by anesthesia providers.

Participants rated the usefulness of apps in various categories on a scale of 0–100. The dosage/pharmaceutical apps scored the highest with a mean score of 78.73 (Figure 4). *Epocrates* was cited as the most commonly used app by anesthesiologists (Figure 5). Other commonly used apps mentioned included apps for *Anesthesiology, Society of Critical Care Medicine (SCCM), UpToDate, Journal of Cardiothoracic and Vascular Anesthesia (JCVTA), Anesthesia & Analgesia, Journal of Pain, QxMD, New England Journal of Medicine (NEJM), ICU Trials, Docphin,* and *BrowZine*.

## 5. Discussion

The history of medical apps is quite brief, and so, its usage has grown exponentially in the last 5 years. Applications or apps, available either in the Apple store or in the Google play store, are programs that are developed to run on smartphones for a specific purpose [5]. Medical apps provide almost everything a textbook can provide with the benefit of being available at one's fingertips. It is not surprising that users are more likely to refer to the convenient and regularly updated medical apps compared to hard copies of textbooks and journals [23]. Additionally, some apps are more user-friendly and faster, while offering the same functionality as the web application [15]. This not only makes possible the realization of integrating technology with clinical practice but also uses the technology as a means to augment learning. A wide variety of apps are available to cater the different needs of the health care professional. The ability to assist professionals at the point of care distinguishes these medical apps from desktop applications. The rampant surge in the use of smartphone apps has led to the coining of a new term “mHealth or mobile health,” which is the use of mobile phones and other wireless technologies in medical care [24]. In addition to apps for references, dosage calculators, and information on symptoms and diagnosis, there are apps that can simulate surgical and anesthetic procedures or conduct simple medical exams, such as hearing and vision tests [9, 14, 19].

One particular area of interest is the use of apps as a clinical tool in integrating technology with clinical practice. With app developers getting more creative every year, the scope for improvement knows no bounds. It will not be long before we see apps being used as a mode of communication within the hospital. In Toronto's Mount Sinai Hospital, the iPhone has been fully integrated into the hospital's daily operations. They have put in place an in-house iPhone application that provides doctors with secure access to patient information [25]. Their in-house application allows doctors to access patient's data securely both within and outside the hospital and thus enable quicker informed decision [26]. Similarly, Samsung Medical Center in Korea allows doctors to access patient information within the hospital through their app Dr. *SMART S* [27].

In this age of smartphones, it was hardly surprising to see that more than 99% of the participants had access to anesthesia apps in our survey. Anesthesiology attendings and residents formed the largest groups, followed by CRNAs. Both in the Apple store and in the Android market, some apps are free to download, while others are available for purchase. It is interesting to note that the price of an app could be a factor in deciding which app someone chooses; only 20% were willing to pay for an app that was $10 or more. Some apps can be downloaded for free but will be fully functional only after a subscription payment [28].

Apps can be advantageous especially when compared to textbook purchase, even if an initial subscription is required. The subscribers may receive updates annually on their app and thus avoid the need to buy newer editions of the hardcover [28]. Commonly used apps among anesthesiologists were categorized as journal apps, educational, airway simulators, dosage/ pharmaceutical apps, conference, calculators, textbooks/references, and board review. Apps providing information on the dosage of drugs and calculators were the most popular and were rated the most useful in their practice in our survey. Frequently used drug reference apps include *Epocrates*, *Skyscape*, *Micromedex*, *FDA drugs*, and *DrugDoses* [8, 19, 29].

The convenience and the ease with which these apps help health care providers at the point of care have made possible the integration of technology with clinical practice. In our survey, nearly one-fourth of the anesthesia providers used apps on a daily basis and more than half did use them at least once a week. Studies have shown that personal digital assistants (PDAs) help physicians make quicker decisions with improved accuracy, efficiency, and positive patient outcomes with reduced adverse events and length of hospital stay [30, 31]. As the later PDAs had internet connectivity and could run applications, it is inferred that the same principle can be applied to the use of smartphones [31]. Despite the popularity of smartphones and apps, we found that 11% of the responders never used apps. One may assume that people of the older age group contributed to these data, but studies have shown that the use of apps was equally distributed among the various age groups [32]. We suspect that this could be related to the lack of reliability of these apps and the perception that they may not be safe.

Our survey clearly illustrates the convenience of apps for health care professionals at the point of care due to portability and quicker access to information. The worrying side to this new trend is the authenticity of some of these apps, with many of them not being reviewed and the absence of a regulatory body. Some of these apps, like opioid dosage conversion apps, were not reliable and accurate, lacked information on evidence-based content, and had no peer reviews in some cases, compromising patient safety [33]. These issues could be some of the reasons why 16% of the respondents in our survey felt that they do not need more apps in their practice, indicating an apparent lack of trust in mobile apps. However, with the rapid growth of the mobile app technology and the realization that apps will play an integral part in patient care, it is vital that the apps be regulated and peer reviewed [34, 35]. It is concerning to notice the lack of professional medical involvement in the development of the app. Some studies have reported that less than 35% of the medical apps had involvement of medical experts during development [13]. In a review of pain management apps, 86% of the apps had no professional involvement, thus increasing the risk for patients being misled [36]. Many apps lacked reviews, and this makes it difficult for the potential buyers to assess the accuracy of apps. Various models and guidelines for app regulation have been proposed, providing some protection against hazardous medical apps for patients and health care professionals [37–39]. It is, however, important to remember that apps are meant only to be an adjunct to clinical acumen and that practitioners must understand the benefits, limitations, and risks associated with apps while making clinical decisions [37]. The fact that some apps lacked medical professional involvement creates a dilemma over their quality and content. There is increasing call for medical apps to be given accreditation, and the user must consider the reliability of the source and content to ensure safety of the patient [34].

The probable solution is to have these apps peer reviewed and regulated. Under the current US FDA guidelines, only those apps which directly influence patient treatment are regulated, leaving the other medical apps unevaluated [40]. Also, the lack of updates in some apps raised the question of accountability of the app developers. To counter this, Cochrane Reviews introduced *iMedicalApps.com*, which is a reputable source for reviews on mobile health applications [41]. While guidelines to regulate the apps are still to take effect, health care professionals can evaluate the apps available before applying them to clinical practice. First, it would be necessary to consider the source of information: whether the developer is a private individual, a health care provider, or professional organization; whether potential conflicts of interest are disclosed; whether references to support content are included in the app; and whether the apps are updated on a regular basis [28]. It is also important to identify the sponsors; an app marketed or sponsored by a pharmaceutical company may be biased towards a particular treatment or pharmaceutical product [28]. Encouraging involvement of anesthesiologists in app development is of no doubt paramount in ensuring patient safety and making sure that users get reliable medical information.

During our search, we found that there were very few apps currently targeting the patients or caregivers. “*Anesthesia Guide for Patients*” is an app available on the Apple store that was developed by an anesthesiology resident for the patient, highlighting different aspects of the perioperative care, including the anesthetic procedure and risks and benefits of different types of anesthesia. However, the app was criticized for not being visually appealing nor interesting, and it was technologically inadequate despite having all the right information [42]. Apps provide information on various disease processes that could influence the anesthetic care, and concerns with the pediatric and obstetric population are some of the areas that app developers could work on [42]. Newer apps could allow the patient to input investigations and upload images and send to the physician.

One limitation of our study is that it only sampled academic anesthesia providers. While it may be reasonable to speculate that anesthesia providers overall have a similar pattern of mobile app utilization, these results cannot be expanded to anesthesiologists in general. Also, it should be highlighted that, in this paper, we primarily aimed to study the utilization of the medical apps by the anesthesia providers; however, whether the utilization of the said app has a meaningful impact on clinical practice of the anesthesiologist is difficult to gauge in this questionnaire study. We anticipate that this study will provide the basis on which further such research can be designed.

## 6. Conclusion

The potential of apps to improve the practice of medicine at the point of care has earned the moniker of “pocket brain.” The use of mobile phone apps is not just limited to reiterating information found in textbooks but also provides a myriad of opportunities to further the ever-changing field of anesthesiology. While it is safe to say that anesthesia apps are here to stay, it is important to keep in mind that they cannot be a substitute for a fully trained anesthesiologist but to merely facilitate the learning and patient care process.

## Figures and Tables

**Figure 2:**
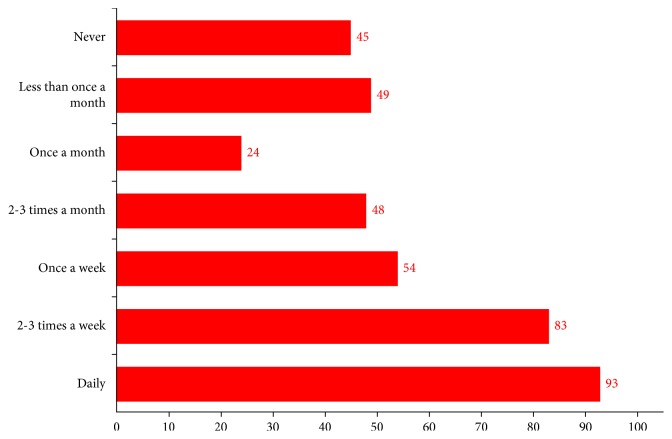
Frequency of use.

**Table 1:** : Survey questions.

(1) Please select your level of training
** **(i) Medical student
** **(ii) Anesthesiology resident
** **(iii) Anesthesiology fellow
** **(iv) CRNA
** **(v) SRNA
** **(vi) Anesthesiology attending
(2) Please select the best answer regarding your subspecialty of training
** **(i) General anesthesiology
** **(ii) Outpatient anesthesiology
** **(iii) Obstetric anesthesiology
** **(iv) Cardiovascular anesthesiology
** **(v) Neuroanesthesiology
** **(vi) Transplant anesthesiology
** **(vii) Pain management
(3) Is your primary mobile phone a “smartphone”, that is, capable of running apps?
** **(i) Yes
** **(ii) No
(4) What operating system does your smartphone run on?
** **(i) Android OS
** **(ii) iPhone OS
** **(iii) BlackBerry OS
** **(iv) Windows mobile OS
** **(v) Symbian
** **(vi) Palm OS
(5) How often do you use smartphone apps related to anesthesiology in your practice?
** **(i) Never
** **(ii) Less than once a month
** **(iii) Once a month
** **(iv) 2-3 times a month
** **(v) Once a week
** **(vi) 2-3 times a week
** **(vii) Daily
(6) Please name some of the apps that you currently use in your anesthesiology practice
** **(i) Epocrates
** **(ii) Oxford American Handbook of Anesthesiology
** **(iii) Sota Omoigui's Anesthesia Drugs Handbook
** **(iv) Anesthesia Drugs
** **(v) Handbook of Clinical Anesthesia
** **(vi) Anesthesia Drips
** **(vii) Memory Master for Nurse Anesthetist
** **(viii) SonoAccess
** **(ix) Hadzic's Peripheral Nerve Blocks
** **(x) Anesthesia 411
** **(xi) Medscape
** **(xii) Regional Anesthesia Assistant
** **(xiii) Block Buddy
** **(xiv) EKG apps
** **(xv) Anesthesia Central
** **(xvi) Anesthesiology Calc
** **(xvii) myAnesthesia
** **(xviii) ASRA Coags
** **(xix) Journal apps (please specify)
** **(xx) Others (please specify)
(7) Please rate the usefulness of various categories on a scale of 0–100
** **(i) Journal apps
** **(ii) Patient education
** **(iii) Airway management simulators
** **(iv) Dosage/pharmaceutical apps
** **(v) Professional society apps/conference adjuncts
** **(vi) Medical calculators
** **(vii) Textbooks
** **(viii) ITE/board reviews
** **(ix) Other educational apps
(8) How much would you be willing to pay for an app that saves you 5–10 minutes/day or 30 minutes/week in your clinical practice?
** **(i) Less than 2 USD
** **(ii) Less than 5 USD
** **(iii) 5–10 USD
** **(iv) 10–25 USD
** **(v) 25–50 USD
** **(vi) More than 50 USD
(9) Would you like to see more “anesthesia-related apps”?
** **(i) Yes
** **(ii) No

**Table 2:** 

Number	Name of the category	Google play store	Apple store	Windows store
1	Journal apps	1	5	None
2	Patient education	2	None	None
3	Airway management simulators	1	5	None
4	Dosage and pharmaceutical apps/medical calculators	20	30	1
5	Professional society apps/conference adjuncts	10	11	2
6	Textbooks/references	57	69	1
7	Board review	22	27	1
8	Other educational apps	28	14	1
	Total	141	161	6
